# Use of MicroRNA Let-7 to Control the Replication Specificity of Oncolytic Adenovirus in Hepatocellular Carcinoma Cells

**DOI:** 10.1371/journal.pone.0021307

**Published:** 2011-07-21

**Authors:** Huajun Jin, Saiqun Lv, Jiahe Yang, Xiaoning Wang, Huanzhang Hu, Changqing Su, Chengliang Zhou, Jiang Li, Yao Huang, Linfang Li, Xinyuan Liu, Mengchao Wu, Qijun Qian

**Affiliations:** 1 Eastern Hepatobiliary Surgery Hospital, Second Military Medical University, Shanghai, China; 2 School of Bioscience and Bioengineering, South China University of Technology, Guangzhou, Guangdong Province, China; 3 College of Life Sciences, Zhejiang Sci-Tech University, Hangzhou, Zhejiang Province, China; University of Hong Kong, Hong Kong

## Abstract

Highly selective therapy for hepatocellular carcinoma (HCC) remains an unmet medical need. In present study, we found that the tumor suppressor microRNA, let-7 was significantly downregulated in a proportion of primary HCC tissues (12 of 33, 36.4%) and HCC cell lines. In line with this finding, we have engineered a chimeric Ad5/11 fiber oncolytic adenovirus, SG7011^let7T^, by introducing eight copies of let-7 target sites (let7T) into the 3′ untranslated region of E1A, a key gene associated with adenoviral replication. The results showed that the E1A expression (both RNA and protein levels) of the SG7011^let7T^ was tightly regulated according to the endogenous expression level of the let-7. As contrasted with the wild-type adenovirus and the control virus, the replication of SG7011^let7T^ was distinctly inhibited in normal liver cells lines (i.e. L-02 and WRL-68) expressing high level of let-7 (>300 folds), whereas was almost not impaired in HCC cells (i.e. Hep3B and PLC/PRF/5) with low level of let-7. Consequently, the cytotoxicity of SG7011^let7T^ to normal liver cells was successfully decreased while was almost not attenuated in HCC cells *in vitro*. The antitumor ability of SG7011^let7T^ *in vivo* was maintained in mice with Hep3B xenograft tumor, whereas was greatly decreased against the SMMC-7721 xenograft tumor expressing a high level of let-7 similar with L-02 when compared to the wild-type adenovirus. These results suggested that SG7011^let7T^ may be a promising anticancer agent or vector to mediate the expression of therapeutic gene, broadly applicable in the treatment for HCC and other cancers where the let-7 gene is downregulated.

## Introduction

Hepatocellular carcinoma (HCC) is the fifth most common cancer worldwide and the third most common cause of death from cancer, resulting in more than 600,000 deaths each year [1]. As surgical techniques have progressed, hepatic resection has evolved into a safe procedure with low operative mortality at large centers [2]. However, no more than 20% of patients with HCC have opportunity to undergo surgery procedures. Chemotherapy and radiotherapy are beneficial complementarities for HCC treatments, yet they lack of tumor specificity and have limited efficacy to the majority of HCC patients at an advanced stage [3]. Thus, it is of importance to seek cancer-specific therapeutic targets and develop effective alternative approaches to specifically treat against HCC.

MicroRNAs (miRNAs) are versatile, noncoding RNAs, which exert posttranscriptional regulation by targeting mRNAs through specific recognition of short sequences, leading to decreased protein production [4]. It has been well documented that miRNA is expressed in tissue- and differentiation state-specific patterns and plays an important role in the control of mammalian growth and development [5]. The let-7 miRNA is one of the first known microRNAs, originally discovered in the nematode *Caenorhabditis elegans*, where it regulates cell proliferation and differentiation [6]. Subsequent work has shown that let-7 is highly conserved across species and found abundantly and ubiquitously expressed in mammalian cells [7]. Conversely, increasing evidence has revealed that let-7 is deregulated in various cancer cells, such as colorectal cancer [8], lung cancer [9], colon cancer [10], ovarian cancer [11], breast cancer [12], gastric cancer [13], malignant melanoma [14], Burkitt lymphoma [15], and acute lymphoblastic leukemia [16]. Recently, it have been reported that members of let-7 family are downregulated in hepatocellular carcinoma [17]–[20].

Given its characteristic in cancer cells, let-7 might serve as an ideal candidate or tool for cancer therapy [21]. Previous studies have validated that *in vitro* or *in vivo* let-7 restorations can inhibit tumor growth [22]. However, like other miRNA replacement or antagonizing therapies, it remains a challenge to effectively deliver the let-7 into cancer cells *in vivo* [23]. As an alternative, the miRNA system is also possible to be used as a tool to control the tropism of oncolytic viruses, yielding safer and more effective anticancer virotherapeutics [24]. Due to its tissue-specific expression profile and short targeting site, miRNA provides considerable flexibility in the design of conditionally replicative viruses. By introduction of miRNA target sequence into the 3′-untranslated region (UTR) of a key gene that has an essential role in viral growth and replication, the virus replication can be controlled by the tissue-specific endogenous miRNA, thus eliminating the unwanted pathology of wild-type virus [25]–[28].

There are currently four oncolytic viruses in phase III trials. Experts in the field believe that an approved product of this new kind of therapeutics is on the horizon [29]. Oncolytic adenovirus holds great promise in anticancer virotherapeutics because of its inherent ability to directly destroy cancer cells and mediate effective transgenic expression in the process of viral production [30]. Previously, diverse schemes have been developed to engineer specificity and improve safety of virotherapy strategies with oncolytic adenovirus, among which are involvement of the E1B deletion [31] or E1A mutation [32], introduction of tumor- or tissue-specific promoters to control the expression of E1A or/and E1B [33]–[35], utilizing liver-specific miR-122a to regulate E1A expression [27], and genetic modification of the adenoviral capsid to change the adenovirus tropism [36]–[38]. Unfortunately, systemic administration of adenoviruses can cause significant infection of hepatocytes and may lead to liver toxicity [39]. Therefore, it remains a challenge to selectively target the cancer cells and leaving the normal cells, especial normal liver cells unharmed in the therapeutic use of oncolytic adenovirus for HCC.

In this study, we detected the expression of let-7 family in a set of primary HCC tissue and HCC cell lines. In line with the finding that let-7 is downregulated in a considerable proportion of primary tissues (36.4%) and HCC cells, we have demonstrated the possibility of using let-7 to fine-tune the replication specificity of a chimeric Ad5/11 fiber adenovius, thereby reducing Ad's hepatotoxicity while maintaining its therapeutic replication within HCC cells.

## Results

### Downregulation of Let-7 in Primary HCC Tissues and HCC Cell lines

Expression of let-7a, a member of let-7 family, in 33 primary HCC tissues was measured by SYBR Green Real-time RT-PCR. Most samples were collected from patients suffered cirrhosis (22/33, 66.7%) and had positive HBsAg (20/33, 60.1%). The results indicated that let-7a expression was decreased in 12 (36.4%, >2 folds) HCC samples compared to adjacent normal liver while increased in 6 (18.2%, >2 folds), and showed no significant difference in other 15 (45.4%, <2 folds). Furthermore, the let-7a was decreased more than 5 folds in 10 samples (30.3%) and decreased more than 20 folds in 4 samples (12.1%). However, no data was revealed that let-7a expression was correlated with the age, gender, tumor size, Hepatitis B infection, or cirrhosis of the HCC patients (Table 1).

**Table 1:** Univariate Analysis of let-7a Expression and Clinicopathological Risk Factors in Patients with HCC.

		Total	relative let-7 expression^a^			r^b^	P
			≥2	(0.5, 2)	≤0.5		
Age (years)	≥55	15	1	8	6	0.193	0.281
	<55	18	5	7	6		
Gender	male	29	6	14	9	0.295	0.096
	female	4	0	1	3		
Tumor size (cm)	≥5	20	4	11	5	0.142	0.431
	<5	13	2	5	7		
Tumor grade	2	6	0	4	2	0.1	0.581
	3	27	6	11	10		
HBsAg	(+)	20	3	7	10	0.235	0.189
	(-)	13	3	8	2		
Cirrhosis	(+)	22	4	9	9	0.09	0.619
	(-)	11	2	6	3		

a The let-7a expression of primary HCC tissues relative to normal liver tissues.

b The correlation coefficient between types of each clinicopathological risk factors and the three status of let-7a expression (i.e. decreased, increased, or unaltered).

**Figure 1:**
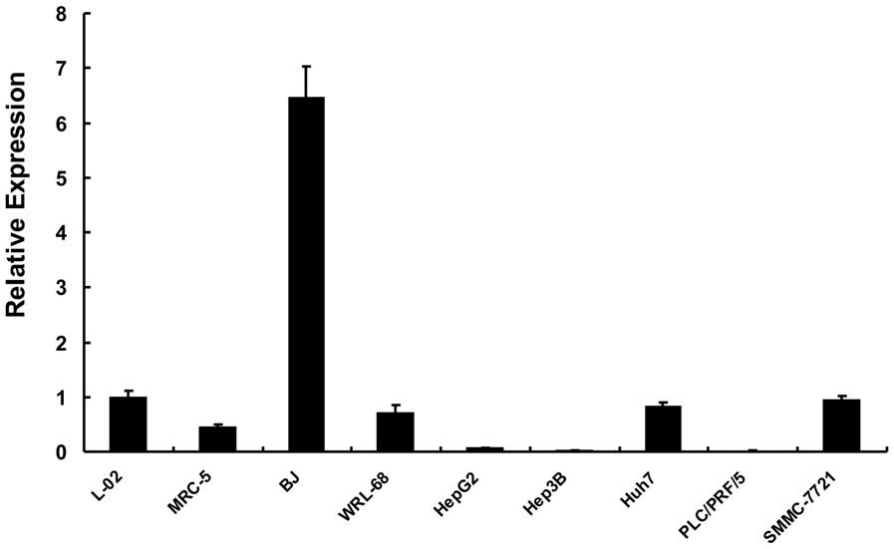
Quantitative measurement of let-7a expression by SYBR Green Real-time PCR. The U6 was used as an endogenous control and L-02 was used as a reference sample. Error bars correspond to mean ± SD. n = 3.

Then we investigated let-7a expression in a series of HCC cell lines. The results showed that let-7a was significantly down-regulated in HepG2 (0.066), Hep3B (0.021), and PLC/PRF/5 (0.010) cell lines as compared with normal liver cell line, L-02 (1.000) and WRL-68 (0.703), while it was slightly down-regulated in Huh7 (0.834) and SMMC-7721 (0.941) cell lines (Fig. 1). The two fibroblast lines (MRC-5 and BJ) showed relative higher level of let-7a expression. Other members of let-7 family (i.e. let-7b, let7c, let-7d, let-7e, let-7f, and let-7g) were also downregulated at some extent in these HCC cell lines. These results indicated that total expression of let-7 family was decreased in a considerable proportion of primary HCC tissues and HCC cell lines.

### Let-7 Target Sites Effectively Regulate the Upstream Gene According to Endogenous Let-7

**Figure 2:**
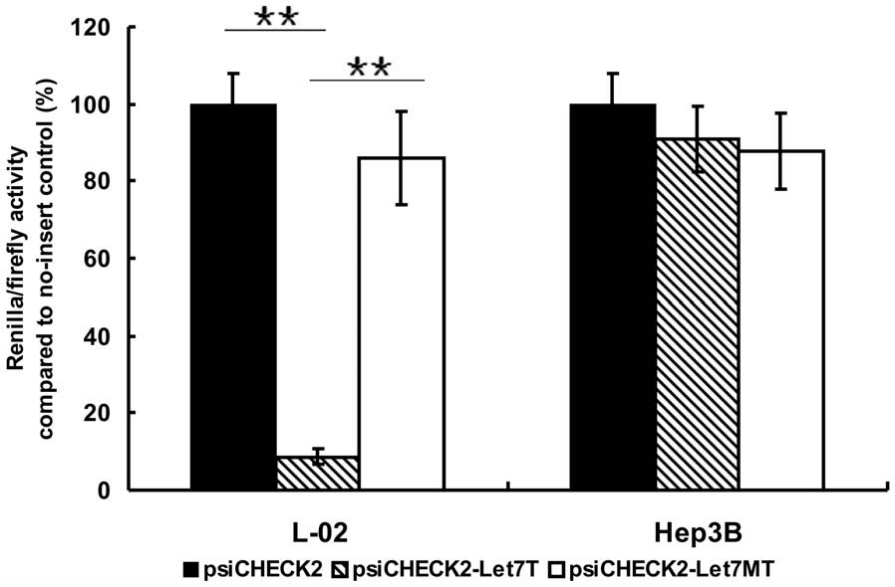
The regulation effect of let-7 target sites on upstream gene, analyzed by dual-luciferase assay system. The relative Renilla/Firefly ratio of cells transfected with the control plasmid, psiCHECK2 was set to 100%. The Renilla/Firefly values of cells transfected with psiCHECK2-Let7T or psiCHECK2-Let7MT was normalized to its corresponding value of cells transfected with psiCHECK2 in a same independent experiment, respectively. Duplicated experiments were conducted from the procedure of cell culture. Error bars correspond to mean ± SD. n = 3. (^**^ P<0.01).

To assess the ability of let-7 in suppressing foreign genes, a let-7-sensitive (psiCHECK2-let7T) (containing eight copies of imperfectly complementary let-7 target sites) and a let-7-insensitive (psiCHECK2-let7MT) (containing eight copies of “seed”-mutated let-7 target sites) luciferase reporter vector was generated and measured the effect of let-7 target sites (let7T) in high (L-02) versus low (Hep3B) let-7-containing cell lines. The results showed that RLuc/FLuc value was 8.7% in psiCHECK2-let7T transfected L-02 cells and 91% in same treated Hep3B cells when individually normalized to that of psiCHECK™-2 transfected cells (Figure 2). By contrast, the RLuc/FLuc value was 86.0% and 87.8% in psiCHECK2-let7MT transfected L-02 and Hep3B cells, respectively (Fig. 2), confirming that the inhibitory effect was indeed mediated by the endogenous let-7. These results demonstrated that eight copies of let-7 target sites could effectively regulate the expression of the upstream gene according to the cellular endogenous expression status of the let-7 gene.

### Cellular Let-7 Controls the Expression of E1A in the Let-7-engineered Adenovirus

**Figure 3:**
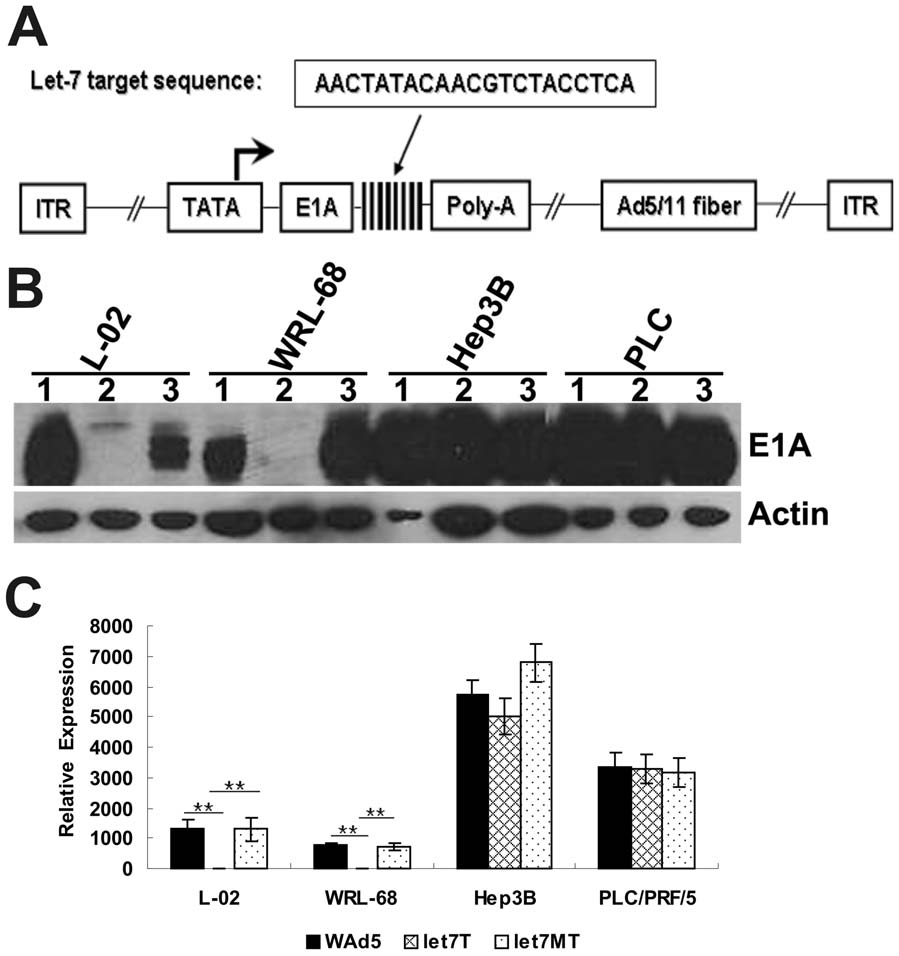
The capacity of let-7 target sites to regulate E1A expression of the recombinant adenovirus, SG7011^let7T^. (A) Virus construction. Eight copies of let-7 target sites were inserted into the 3′UTR of E1A gene and the Ad5 fiber was replaced by Ad5/11 chimeric fiber. ITR, inverted terminal repeats. (B) Detection of E1A protein expression by Western Blotting. The 1, 2, 3 lane represented cells infected with WAd5, SG7011^let7T^ and SG7011^let7MT^, respectively. PLC, PLC/PRF/5. (C) Detection of E1A mRNA expression by quantitative RT-PCR. The gene of GAPDH was used as an endogenous control and HEK293 cell uninfected with adenovirus was used as a reference sample. Let7T, SG7011^let7T^; Let7MT, SG7011^let7MT^. Error bars correspond to mean ± SD. n = 3. (^**^ P<0.01).

Having identified the capacity of let-7 target sites in the regulation of the upstream gene, a let-7-regulated fiber chimeric adenovirus, SG7011^let7T^, was constructed by incorporating the same fragment containing eight copies of let-7 target sites immediately after the stop codon of E1a gene and substituting the Ad5 fiber protein with the Ad11 knob domain (Fig. 3A). At the same time, the fragment containing eight copies of “seed”-mutated let-7 target sites were individually inserted into the same site to generate a control fiber chimeric adenovirus, SG7011^let7MT^.

Western blotting was used to test the ability of let-7 target sites in regulating adenoviral E1A expression in HCC cells and normal liver cells. The results showed that within normal liver cell lines that have high cellular let-7, the E1A protein was dramatically suppressed in the SG7011^let7T^-infected cells, but remained highly expressed in the SG7011^let7MT^- and WAd5-infected cells. In HCC cell lines that have low cellular let-7, however, the E1A protein expressed similarly among the SG7011^let7T^-, SG7011^let7MT^- and WAd5-infected cells (Fig. 3B).

Since let-7 has been documented to mediate mRNA deadenylation via binding on its imperfectly complementary target sites [40], we next measured the amount of E1A mRNA in SG7011^let7T^-, SG7011^let7MT^- and WAd5-infected cells by quantitative RT-PCR. In good agreement with the Western blotting data, E1A mRNA levels in SG7011^let7T^-infected L-02 and WRL-68 cells were significantly lower than those in SG7011^let7MT^- and WAd5-infected corresponding cells, whereas in Hep3B and PLC/PRF/5 cells, the viruses expressed similar amounts of E1A mRNA (Fig. 3C). These results indicated that the negative regulation of cellular let-7 on E1A may through mRNA destruction.

### Replicative Control of Recombinant Adenovirus by let-7 Target Sites

**Figure 4:**
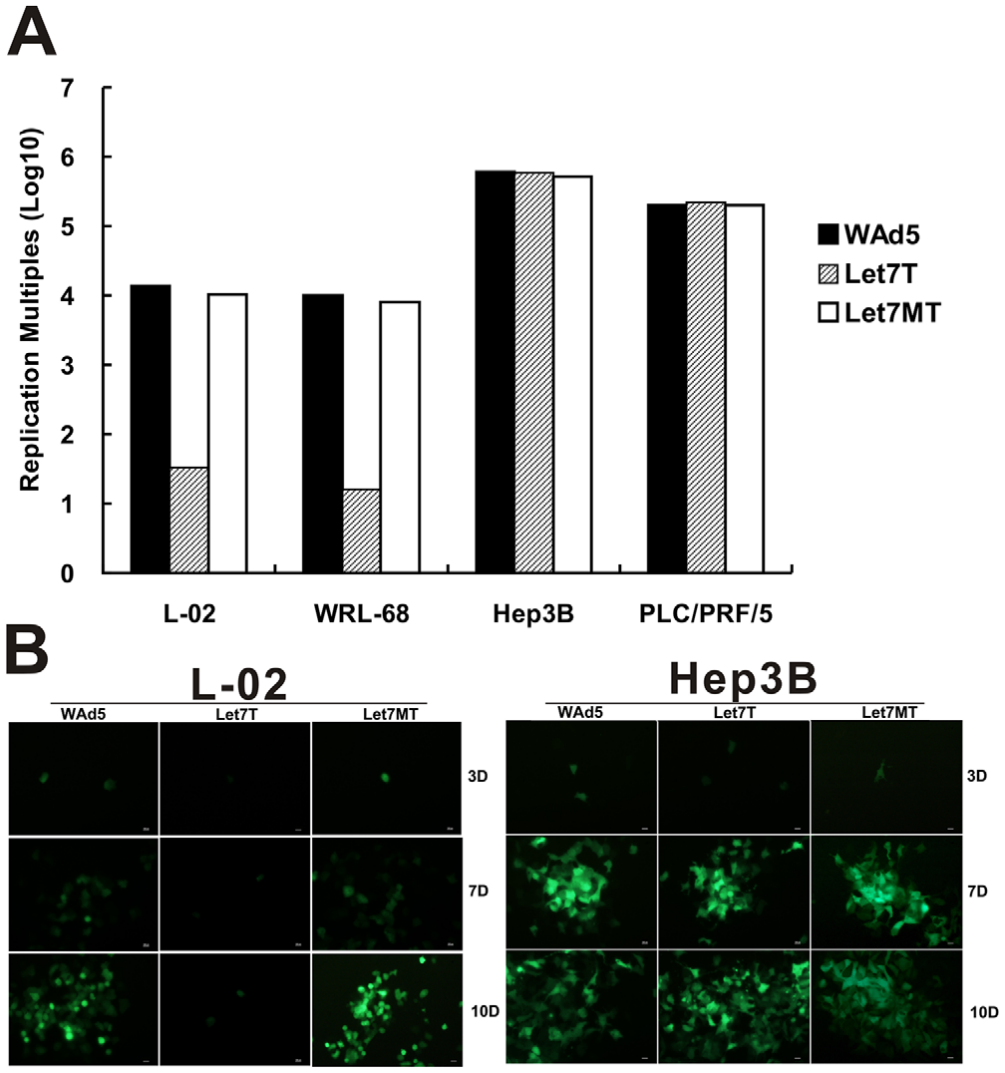
The capacity of let-7 target sites in regulation of virus replication. (A) Viral replication assay. Cells were planted in 6-well dishes (10^6^ cells/well) and infected with indicated viruses at a MOI of 5 pfu/cell, and the cell lysates and supernatant at 48 h post infection were tittered by the TCID50 method in HEK293 cells and normalized to those at the beginning of infection. Let7T, SG7011^let7T^; Let7MT, SG7011^let7MT^. (B) Fluorescence analysis. The L-02 and Hep3B cells were infected with test or control virus at an MOI of 0.01 and 0.001, respectively. Three days (3D), 7 days (7D), and 10 days (10D) post infection, cells were observed under fluorescent microscope and photos were taken. The WAd5, let7T, and let7MT represented WAd5-EGFP, SG7011^let7T^, and SG7011^let7MT^, respectively. Bar = 10 µm.

Viral replication was detected to evaluate the capacity of let-7 target sites in regulating the adenoviral replication in HCC cells and normal liver cells. The results showed that the testing virus, SG7011^let7T^, replicated preferably in HCC cells but significantly decreased in normal liver cells (p<0.01) (Fig. 4A). Conversely, the control virus, SG7011^let7MT^ was replicated in both normal liver cells and HCC cells, showing a viral tropism similar with that of wild-type adenovirus (Fig. 4A). The replication of SG7011^let7T^ decreased at lest 300-fold than that of SG7011^let7MT^ and WAd5 in normal liver cells, whereas almost not attenuated in HCC cells. These results indicated that the introduced let-7 target sites could effectively control adenoviral replication according to the expression of cellular endogenous let-7.

Alternatively, we determined the selective replication of SG7011^let7T^ in normal liver and HCC cells via SG7011^let7T^-EGFP, a recombinant adenovirus carrying EGFP gene. The results observed here were consistent with those from the evaluation of viral replication. In L-02 cells, only a few scattered cells emitted fluorescence 3 days post infection and this status also persisted for 7–10 days since infection (Fig. 4B). However, in the Hep3B cells, the fluorescence emerged from a single cell to many surround cells and simultaneously more and more cells died with EGFP degradation 7 or 10 days post infection. In contrast, in both L-02 and Hep3B cells infected with SG7011^let7MT^-EGFP or WAd5-EGFP, the fluorescence gradually increased and some cells died with EGFP degradation from 3 to 10 days post infection.

### Let-7 Target Sites Reduce Hepatic Toxicity of Recombinant Adenovirus while Remain its Oncolytic Effects on HCC Cells *in vitro*

**Figure 5:**
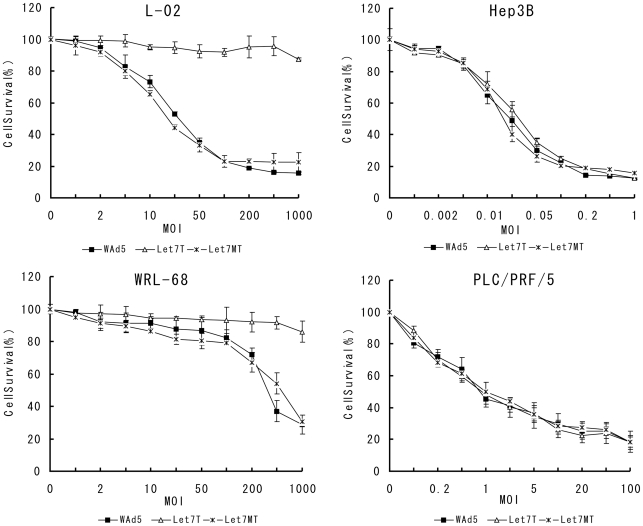
The cytotoxicity of recombinant adenovirus to normal liver cells and HCC cells *in vitro*, analyzed by MTT assay. Cell viability of L-02, WRL-68, Hep3B, and PLC/PRF/5 were infected with WAd5, SG7011^let7T^, and SG7011^let7MT^ at various MOIs were measured on day 7 postinfection by MTT assay. Let7T, SG7011^let7T^; Let7MT, SG7011^let7MT^. Error bars correspond to mean ± SD. n = 3.

Since the engineered adenovirus SG7011^let7T^ was replicated preferentially in tumor cells where the let-7 is downregualted, its cytotoxic effects in normal liver and HCC cells were quantified by 3-(4, 5-dimethylthiazol-2-yl)-2, 5-diphenyltetrazolium bromide (MTT) assay. The results showed that the hepatic toxicity of SG7011^let7T^ was significantly decreased as compared to that of WAd5 (p<0.01) (Fig. 5). Seven days after the SG7011^let7T^ infection at MOI of 1000 pfu/cell, the viability of L-02 and WRL-68 cells remained 87.4% and 86.0%, respectively. In contrast, in WAd5-infected L-02 cells, the cell viability was 52.9% at MOI of 20 pfu/cell, and 15.5% at an MOI of 1000 pfu/cell. In WAd5-infected WRL-68 cells, the cell viability was 37.0% and 28.7% at MOI of 500 pfu/cell and 1000 pfu/cell, respectively. The control virus, SG7011^let7MT^ had similar cytotoxicity to normal liver cells, showing IC50 (MOI values of 50% viability) at 20 and 500 pfu/cells in L-02 and WRL-68 cells, respectively. In HCC cell lines, however, SG7011^let7T^ showed similar oncolytic effect with WAd5 and SG7011^let7MT^. The IC50 of SG7011^let7T^, WAd5, and SG7011^let7MT^ was all between 0.01 and 0.05 pfu/cell in Hep3B cells, and between 0.5 and 1.0 pfu/cell in PLC/PRF/5 cells.

### Introduction of Let-7 Target Sites does not Attenuate Oncolytic Effects of Recombinant Adenovirus on HCC Cells *in vivo*

**Figure 6:**
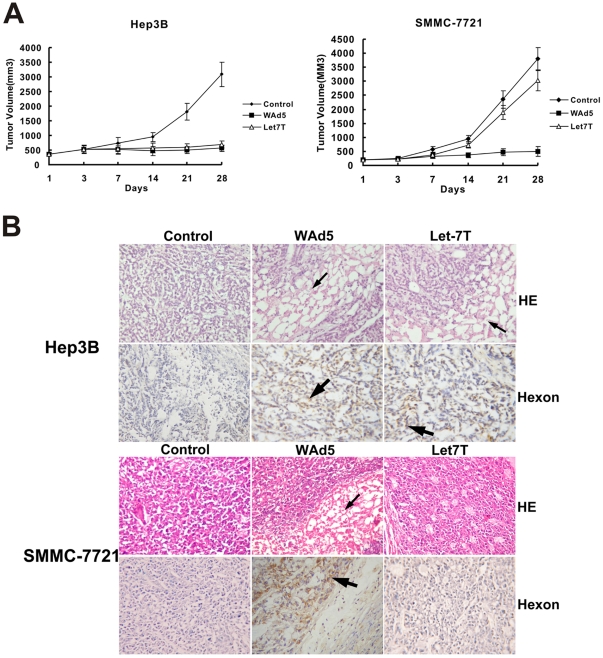
Potent antitumor activity on hepatocellular carcinoma xenografts with SG7011^let7T^. (A) Measurement of antitumor activity. BALB/c nude mice (n = 10 per group) bearing Hep3B or SMMC-7721 xenografts were treated with WAd5, SG7011^let7T^, and virus preservation buffer control by i.t. administration five times at a dosing volume of 100 µl (5×10^8^ pfu), on the days of 1, 3, 5, 7 and 9 following the initiation. Tumor volume was estimated with the formula: (maximal diameter)×(perpendicular diameter) 2×0.5. Error bars correspond to mean ± SD. (B) Pathologic examination of Hep3B or SMMC-7721 tumor xenografts. The necrosis areas in HE staining was indicated by thin arrow and the area showing positive signal in immunohistochemistry staining with anti-hexon antibodies was indicated by bold arrow. (×400) Let7T, SG7011^let7T^.

The antitumor efficacy of SG7011^let7T^ as a monotherapy was evaluated in two kinds of s.c. xenograft HCC tumor model (i.e. Hep3B and SMMC-7721). In Hep3B xenografts model, a tumor model with low level of let-7, significant antitumor efficacy was observed. By day 28, the placebo treated tumors had increased 10-fold in volume whereas the SG7011^let7T^ treated tumors as a concentration of WAd5 pfu per dose grown a little in size (1.6 fold of the day 1 volume) (Fig. 6A). These results indicated that the antitumor effect of SG7011^let7T^ was closed to that of WAd5 in Hep3B xenografts models. Conversely, in the SMMC-7721 xenografts models, a tumor model containing let-7 at a level corresponding to normal hepatocytes, almost no antitumor effect was displayed in the SG7011^let7T^ treatment. The curve of tumor growth in the SG7011^let7T^-treated group was similar with that of placebo control while significantly different from the WAd5-treated one (Fig. 6A), assuming that the cytotoxicity of wild-type adenovirus to normal liver might be eliminated in the engineered adenovirus SG7011^let7T^.

The tumor samples were examined histologically by H&E staining and immunohistochemical staining for hexon protein. For the Hep3B tumor, the cancer cells grew luxuriantly with small foci of necrosis in the control group, whereas many wide areas of necrosis were observed and hexon protein was detected in the both WAd5- and SG7011^let7T^-treated groups (Fig. 6B). For the SMMC-7721 tumor, few necroses were observed in the SG7011^let7T^-treated group and the signal of hexon protein was weak, which was remarkably different from the situation of the WAd5-treated group. These results suggested that the test virus SG7011^let7T^ could efficiently lyse tumor cells with lower level of let-7, leaving normal liver cells with higher level of let-7 spared.

## Discussion

It has been well documented that members of let-7 family play important roles in both normal development and tumorigenesis [41]. In normal stem cells, let-7 can negatively regulate “stemness” by repressing self-renewal and promoting differentiation [21]. Consistent with its role of tumor suppressor, let-7 is found deregulated in various forms of cancers and the decreased expression of let-7 is functionally linked to tumor cell biology, paralleled with the upregulated expression of proto-oncogenes, reflecting poor patient prognosis at least in lung cancers [21]. More interestingly, it has been demonstrated that let-7 is markedly reduced in breast tumor initiator cells, regulating their self-renewal and tumorigenicity [42]. Here we reported that let-7 was downregulated (>2 folds) in a proportion of primary HCC tissues (12/33, 36.4%), most which were collected from patients had a history of HBV infection and suffered cirrhosis (Table 1). Moreover, ten HCC tissues (10/33, 30.3%) even had deregulated let-7 which was more than 5 folds lower relative to their corresponding normal liver tissues. This result was consistent with the finding from recent work via deep-sequencing, showing that the abundance of several members of let-7 family were among the top 10 most abundantly expressed miRNAs in normal livers and the abundance of let-7 remarkably decreased (>10 folds) in a considerable proportion of HCC tissues as compared with matched normal liver tissues. To date, however, the underlying mechanism for the repression of let-7 in HCC remains unknown. The upregulation of LIN28B [43], [44], a suppressor of let-7 expression, may be one of possible causes. Furthermore, since the constitute of HCC tissue is highly heterogeneous, it is unclear if the lower levels of let-7 in HCC tissue vs. normal liver tissue were due to transformation of hepatocytes or differences in the relative abundance of other cell types in these paired tissues specimens.

The strategy using tissue-specific microRNAs to modify virus tropism has been exploited in several types of oncolytic virus [24]. To adenovirus, the liver-specific miR-122a has been introduced to regulate the expression of E1A and succeed to decrease hepatotoxicity of wild-type adenovirus [28]. Taken together the data presented here, it indicates that microRNA-based regulation strategy alone is effective to control the adenovirus tropism. Furthermore, due to its specific insertion sites (3′-untranslated region) and special regulation stage (post transcription), the strategy can also be combined with other virus regulation approach, such as promoter control, deletion/mutation control, and capsid modification. In fact, as an example, the miR-122a-meadiated regulation via three miR-122a target sites downstream E1A gene and another regulation approach based on CR2-deletion targeting to Rb-deficient cancer cells has been successfully combined to fine-tune oncolytic adenoviruses [27]. Moreover, this strategy introduces no mutation in virus protein, leaving virus packaging efficiency unharmed and thus without attenuation of adenovirus's ability to kill cancer cells. Such a view has been repeatedly verified in previous two explorations and present study [27], [28]. Hence, the viral regulation strategy based on tissue-specific microRNA (including normal tissue specific-expressed and cancer tissue specific-suppressed) is promising to generate conditionally replicative adenoviruses.

Since let-7 is expressed at lower levels in HCC cells than normal liver cells and can affect either the stability or translation of the target mRNA [40], it is feasible to decrease the Ad's liver tropism via introducing let-7 target sites to regulate E1A expression. As shown above, both mRNA and protein of the E1A was tightly regulated according to cellular let-7. Consequently, the replication of the engineered virus was decreased more than 300-fold compared to the control virus in normal liver cells, whereas the proliferation rate between the engineered virus and the control virus was similar in HCC cells. Accordingly, the cytotoxicity of the engineered virus was distinctly declined in normal liver cells while alomst not impaired in HCC cells. These results indicated that introduction of let-7 target sites downstream of E1A could successfully decrease the hepatotoxicity of wild-type adenovirus without attenuation of its ability to kill HCC cells with lower level of let-7. Thus, the engineered adenovirus fine-tuned by let-7 presented here may serve as a potential anticancer agent or a therapeutic vehicle for harboring antitumor genes, broadly applied in the treatment against cancer with the downregulated cellular let-7, including HCC.

## Materials and Methods

### Patients and Sample Collection

Thirty-three pairs of primary hepatocellular carcinoma (HCC) tissues and corresponding adjacent normal liver tissues were used. All clinical samples were collected with the approval of Ethnics Committee of Eastern Hepatobiliary Surgery Hospital and written informed consent from HCC patients undergoing surgery. Pathological diagnosis was made according to the histology of tumor specimens and examinations were performed by experienced pathologists. All cancer tissue samples were grossly dissected and snap-frozen in liquid nitrogen within 20 min of removal and stored at −80°C until RNA extraction.

### Cell Culture

Human HCC cell lines (HepG2, Hep3B, PLC/PRF/5, Huh7), human embryonic liver cell line WRL-68, fibroblast lines (MRC-5 and BJ), and human embryo kidney cell line (HEK293) were purchased from the ATCC (Manassas, VA). The human HCC cell line (SMMC-7721) and the human normal liver cell line (L-02, also designated as HL-7702) were obtained from the Institute of Cell Biology, Chinese Academy of Sciences (Shanghai, China). All cell lines were cultured according to the instructions of the providers.

### Real-time RT-PCR

Total RNA was isolated using a miRNeasy mini kit (Qiagen, Valencia, CA) and reverse transcriptase reactions were performed using a miScript Reverse Transcription Kit (Qiagen, Valencia, CA). The expression of let-7 family was measured by miScript SYBR Green PCR Kit (Qiagen, Valencia, CA) according to the manufacturers' protocol and normalized to U6 levels. The relative expression values of different cells or samples were determined by using L-02 cell as a reference sample. E1A expression in adenovirus infected cells was measured and quantified by SYBR Green Realtime PCR Kit (ToYobo, Osaka, Japan) according to the manufacturers' protocol, using GAPDH as an endogenous control and HEK293 as a reference sample.

### Preparation of Luc Reporter Plasmids and Luciferase Assays

Eight copies of imperfectly complementary let-7 target sites (AACTATACAACGTCTACCTCA) [45] or “seed”-mutated let-7 target sites (AACTATACAACGTCTTGGAGT) with 2–6 oligonucleotides intervals were synthesized (TAKARA, DaLian, China)and cloned downstream of the Renilla luciferase gene of psiCHECK™-2 (Promega, Madison, WI), a plasmid containing both Renilla luciferase (RLuc) and firefly luciferase (FLuc) reporter gene, to generate reporter plasmids sensitive to let-7 (i.e. psiCHECK2-let7T), and control plasmid insensitive to let-7 (i.e. psiCHECK2-let7MT). L-02 or Hep3B cells in 12-well plates were transfected with 5 ng of psiCHECK-2, psiCHECK2-let7T, or psiCHECK2-let7MT by using Lipofectamine 2000 (Invitrogen, Carlsbad, CA). Twenty-four hours posttransfection, values of relative luciferase activity (RLuc/FLuc) were measured using the dual-luciferase assay system (Promega, Madison, WI). Duplicated experiments were conducted starting from the procedure of cell culture and either RLuc/FLuc value for the psiCHECK2-let7T or psiCHECK2-let7MT constructs was normalized to its corresponding value for the psiCHECK2 constructs in a same independent experiment, respectively.

### Viral Construction

pXC-miR was constructed from adenovirus shuttle plasmid, pXC.1 (Microbix Biosystems Inc., Toronto, Canada) by overlap PCR to introduce one *Bst*BI and one *Sal*I restriction site immediately after the stop codon of E1a gene. And then, DNA fragment containing eight copies of let-7 target sites or seed-mutated let-7 target sites mentioned above was inserted into pXC-miR between *Bst*BI and *Sal*I site to generate pXC-let7T and pXC-let7MT, respectively. pPE11 is a adenovirus packaging plasmid derived from pBHGE3 and pBHGlox DeltaE1.3 (Microbix Biosystems Inc.) constructed by replacing Ad5 fiber with Ad5/F11 chimeric fiber using the same procedure as described in our previous publication [33]. Finally, the plasmids pXC-let7T, pXC-let7MT, and pXC.1 were individually transfected into HEK293 cells using Effectene Transfection Reagent (Qiagen, Valencia, CA) together with pPE11. The obtained chimeric adenovirus was named as SG7011^let7T^, SG7011^let7MT^, and WAd5, respectively.

### Western Blot Analysis

L-02, HepG2, Hep3B, and PLC/PRF/5 cells were seeded in six well plates at a density of 5×10^5^ per well, cultured for 24 h, and infected with viruses at a MOI of 5 pfu/cell. Two days later, cells were harvested and lysed by using M-PER Mammalian Protein Extraction Reagent (PIERCE, Rockford, Ill). Total protein (20 µg) was separated on 10% SDS-polyacrylamide gel and electroblotted onto PROTRAN nitrocellulose transfer membrane (Schleicher & Schuell, Dassel, Germany) and carried out Western blot by using the mouse anti-adenoviral E1a antibody M73 (Santa Cruz Biotechnology, CA) and the horseradish peroxidase (HRP)-conjugated goat anti-mouse IgG (Cell Signaling Technology, Beverly, MA). The signals were visualized with LumiGLO chemiluminescent reagent and peroxide (Cell Signaling Technology).

### Viral Replication Study

Human HCC cell lines (HepG2 and Hep3B) and normal liver cell lines (L-02 and WRL-68) were seeded in six-well plates at 5×10^5^ cells per well and cultured until cancer cells were in log phase and normal cells at contact inhibition stage. Subsequently, the cultured cells were infected with the adenoviruses, SG7011^let7T^, SG7011^let7MT^ and WAd5 at a multiplicity of infection of 5 (MOI = 5). Forty-eight post infection, the cells were harvested and lysed by three cycles of freeze and thaw, and their viral titers were examined with the tissue culture infectious dose 50 (TCID50) methods as described in our previous publication [34]. The titer data at 48 h were normalized to those at the beginning of infection.

### Experiments with Replicative Adenovirus Carrying EGFP gene

An adenovirus packaging plasmid carrying EGFP gene, pPE11-EGFP, was constructed by incorporating the EGFP expression cassette into E3 region of pPE11 genome through Gateway system using the same procedure as described in our previous publication [35]. And then it was homologous recombined with the plasmids pXC-let7T, pXC-let7MT, and pXC.1 individually using standard procedures to generate recombinant adenovirus carrying EGFP, referred to as SG7011^let7T^-EGFP, SG7011^let7MT^-EGFP and WAd5-EGFP, respectively. L-02 and Hep3B cell lines were cultured in 6-well plates at a density of 4×10^5^ cells/well. After 24 h, they were infected with SG7011^let7T^-EGFP, SG7011^let7MT^-EGFP, and WAd5-EGFP respectively at a MOI of 0.01 for L-02 cells and 0.001 for Hep3B. Three days, 7 days, and 10 days post infection, cells were observed under fluorescent microscope and photos were taken.

### Adenovirus Cytotoxicity Assay in vitro

L-02, WRL-68, HepG2, Hep3B was planted at a density of 1×10^4^ in 96-well plates and infected with SG7011^let7T^, SG7011^let7MT^ and WAd5 at a gradient of MOI from 0.001 to 100 pfu/cell. Cell viability was measured by 3-(4, 5-dimethylthiazol-2-yl)-2, 5-diphenyltetrazolium bromide (MTT) assay according to the instructions of Cell Proliferation Kit I (Roche Molecular Biochemicals, Mannheim, Germany).

### Animal Experiments

BALB/c nude mice (nu/nu) were purchased from Shanghai Experimental Animal Center, Chinese Academy of Sciences. All animal experiments were carried out in adherence with the National Institutes of Health Guidelines on the Use of Laboratory Animals and approved by the Second Military Medical University Committee on Animal Care (EC10-055). Mice were injected s.c. in the right flank with 1×10^7^ Hep3B or SMMC-7721 cells in matrigel (injection volume of 100 µl). When the Hep3B tumors reached a mean tumor volume of 450 mm^3^ or the SMMC-7721 tumor reached a mean tumor volume of 200 mm^3^ [volume = (W^2^×L)/2; W, width; L, length, in cubic millimeters], animals with the Hep3B or SMMC-7721 xenografts (n = 10 per group) were randomly distributed into three treatment groups, respectively. The treatment groups were injected intratumorally with SG7011^let7T^ or WAd5 five times at a dosing volume of 100 µl (5×10^8^ pfu), on the days of 1, 3, 5, 7 and 9 following the initiation. The control group was intratumorally injected with the placebo paralleledly [10 mmol/L Tris-HCl (pH 8.0), 2 mmol/L MgCl_2_, 4% sucrose]. Tumor volumes were measured once weekly after the first virus injection on day 1.

### Histology and Immunohistology

For histologic examination, the explanted tumors were fixed in 10% buffered formalin and then 5-µm paraffin sections were used for H&E staining or processed for immunohistochemical analysis. Immunohistochemistry was carried out with anti-adenoviral hexon protein antibody (Biodesign International, Saco, ME) following the protocol of the manufacturer as described previously.

### Statistical Analysis

Data are expressed as the mean of triplicates ± standard deviation unless otherwise stated. Results were compared for statistical significance by applying t-test. Correlation between relative let-7 expression and clinicopathologic parameters was evaluated by using Pearson's chi-square test. Data were considered statistically significant when P<0.05.
